# Evaluation of chromosome 17 polysomy in breast cancer by FISH analysis of whole nuclei, and its clinicopathological significance

**DOI:** 10.3892/ol.2014.2001

**Published:** 2014-03-28

**Authors:** HUIYONG JIANG, XIAOYAN BAI, FANJUN MENG, CHENG ZHANG, XUEFENG ZHANG

**Affiliations:** 1 Department of General Surgery, General Hospital of Shenyang Military Area Command, Shenyang, Liaoning 110840, P.R. China; 2 Division of Nephrology, Guangdong Provincial Institute of Nephrology, Nanfang Hospital, Southern Medical University, Guangzhou, Guangdong 510515, P.R. China; 3 Department of Gastroenterology, No. 202 Hospital of People’s Liberation Army, Shenyang, Liaoning 110003, P.R. China

**Keywords:** breast cancer, nuclei microarray, fluorescence *in situ* hybridization, HER2 gene

## Abstract

Human epidermal growth factor receptor 2 (HER2) amplification and overexpression are associated with poor prognosis and resistance to cytotoxic drugs in patients with breast cancer. Increases in the number of HER2 gene copies have been shown to be associated with chromosome 17 polysomy. The use of whole, intact nuclei for fluorescence *in situ* hybridization (FISH) assay improves the accuracy of the results. FISH analysis of whole nuclei (WNFISH) and immunohistochemistry (IHC) were used to analyze HER2 gene amplification and HER2 protein expression in 109 breast cancer specimens. Chromosome 17 polysomy and its correlations with HER2 gene amplification, HER2 protein expression and the clinicopathological outcomes of the patients were also investigated. Among the 109 cases, WNFISH detected HER2 amplification in 30 cases, equivocal amplification in 19 cases and no amplification in 60 cases. WNFISH detected chromosome 17 centromere (CEP17) polysomy in 37 cases and no polysomy in 72 cases. Among the 109 cases assessed by tissue microarray and IHC, 31 cases were HER2-negative, 14 cases were scored 1+, 23 cases were scored 2+ and 41 cases were scored 3+. The results demonstrated that in the cases with chromosome 17 polysomy, the HER2 gene was amplified, HER2 protein expression was increased and the incidences of nuclear atypia and lymph node metastases were higher compared with those in the cases without chromosome 17 polysomy. Chromosome 17 polysomy may correlate with increased malignant potential and metastatic spread in breast cancer.

## Introduction

Breast cancer is one of the common types of cancer in females. The incidence of breast cancer increases each year, and the proportion of affected young females also increases, posing a serious threat to the health of the population (1). Overexpression of the human epidermal growth factor receptor 2 (HER2) gene has been confirmed to closely correlate with the prognosis of breast cancer patients and with the effects of chemotherapy and hormonal therapy. The HER2 gene encodes a 185-kDa transmembrane receptor with tyrosine kinase activity, without a known ligand (2). HER2 is involved in the regulation of cell growth, survival and differentiation. Based on the definition by the American Society of Clinical Oncology and College of American Pathologists (ASCO/CAP) (3), it is estimated that ~14.7% of breast carcinomas exhibit HER2 genetic heterogeneity (4). For the assessment of the HER2 gene copy number, it has been suggested that systems assessing the HER2/chromosome 17 centromere (CEP17) ratio, such as dual- or single-color fluorescence *in situ* hybridization (FISH) or chromogenic *in situ* hybridization (CISH), provide a more accurate evaluation of HER2 amplification than single-probe systems. These methods have successfully identified patients who will benefit from trastuzumab therapy in clinical trials (5). Approximately 8% of breast cancers exhibit increased copy numbers of CEP17 using FISH (i.e., average CEP17 >3.0 per nucleus), and these cancers possess chromosome 17 polysomy (3,6,7).

Abnormalities of chromosome 17 are important molecular genetic events in tumorigenesis, particularly in breast cancer (8). Several important genes, including the oncogenic genes HER2 and *TOP2A* and the tumor suppressive gene p53, are essential in the development and progression of breast cancer (9). The polysomy of chromosome 17, one of the major types of abnormality of chromosome 17, is frequent and identified in 20–40% of invasive breast carcinomas (10). An increased HER2 gene copy number has been reported to be associated with polysomy 17, a contributing factor for HER2 protein overexpression (11,12). Studies have also shown that chromosome 17 polysomy may be associated with HER2 gene expression, the prognosis of breast cancer patients and sensitivity to chemotherapy (13). However, several other studies identified no effects of polysomy 17 on HER2 protein expression (10), or found that only a small number of cases were affected (14,15). If polysomy 17 is an effective factor influencing immunohistochemistry (IHC) scores, a number of positive IHC cases caused by the polysomy may be missed when following the algorithm for FISH analysis (3). As a result, an accurate assessment of HER2 status and chromosome 17 polysomy is of great importance when identifying patients who are eligible for trastuzumab therapy (16,17).

In the majority of studies, thin paraffin tissue sections (4 μm) have been used for FISH assay, which is defined as a routine procedure in the International Standard Guide for analyzing HER2 amplification and chromosome 17 polysomy (3). However, the average diameter of a nucleus is >6 μm and the diameter of a tumor cell nucleus is often much greater. It may be assumed that, due to tissue sectioning, nuclei are not intact in 4-μm-thick sections, causing a possible loss of some genetic material. This may lead to inaccurate results, particularly in the evaluation of polysomy 17 by gene copy numbers using FISH, as a number of copies may be in an adjacent section. As a result, using whole, intact nuclei for FISH may improve the accuracy of the results.

In the present study, FISH analysis of whole nuclei (WNFISH) and IHC were used to analyze HER2 gene amplification and HER2 protein expression in 109 breast cancer specimens.

## Materials and methods

### Case selection

One hundred and nine patients (aged 33 to 83 years; median, 49.5 years old) with invasive breast ductal carcinoma who were treated at the General Hospital of Shenyang Military Area Command (Shenyang, China) between 2010 and 2011 were selected for this retrospective study. HER2 gene status was evaluated in the 109 formalin-fixed paraffin-embedded (FFPE) tissues by WNFISH with nuclei extracted from the FFPE tissue blocks. The study was approved by the Ethics Committee of the General Hospital of Shenyang Military Area Command. Written informed consent was obtained from all patients according to the instructions of the Ethics Committee of the General Hospital of Shenyang Military Area Command.

### Preparation of whole nuclei

A tissue microarray (TMA) was constructed using the FFPE breast cancer tissue blocks. For each specimen, four tissue cores were collected using a blunt core needle, 0.6 mm in diameter (18). The needle was a component of a Manual Tissue Arrayer Beecher Instruments Inc., Silver Spring, MD, USA) and was pushed through the entire paraffin tissue block. The cut tissue core remaining in the needle was then forced into a 1.5-ml polypropylene microcentrifuge tube.

Xylene (1 ml) was added into the tube twice, each time for 20 min with light agitation. Subsequently, 1.0 ml dehydrated ethanol was added twice, each time for 3 min with light agitation. Then, 80%, 70% and 50% ethanol (1 ml of each) was successively added into the tube, each for 3 min with light agitation. The ethanol was discarded, and the tube was dried at 45°C for 10 min to evaporate the remaining ethanol. Enzymatic digestion was then performed by adding 300 μl freshly prepared proteinase K solution [0.01% proteinase K, 30 mAnson-U/mg, in 0.05 mol/l Tris(hydroxymethyl)aminomethane hydrochloride (pH 7.0), 0.01 mol/l ethylenediaminetetraacetic acid disodium salt and 0.01 mol/l sodium chloride] purchased from Merck KGaA (Darmstadt, Germany) to the microcentrifuge tube. The sample was then incubated at 37°C for 2 h. To aid the enzymatic digestion, the sample was vortexed for 3 sec at 20-min intervals during this incubation period.

The solution was mixed and centrifuged at 300 × g for 5 sec to deposit the tissue mass. The suspension containing the nuclei was sampled using a pipette. The nuclei were washed by resuspension and vortexing in 100 μl phosphate-buffered saline (PBS). The PBS solution was removed and the nuclei were fixed by resuspension and vortexing twice in freshly prepared fixative (three parts methanol and one part glacial acetic acid). The fixative was removed and the nuclei were resuspended in 100 μl distilled water. The nuclei density was calculated on a cell counting plate and adjusted to 1×10^4^ cells/μl using distilled water.

The pretreated nuclei suspension (1×10^4^ cells/μl) was pipetted onto poly-L-lysine-coated slides (Shanghai Baoman Biotechnology Co., Ltd., Shanghai, China). The slides were heated at 65°C for 1 h, and the nuclei were then ready for the consecutive experiments after air drying.

### WNFISH

WNFISH analysis was performed using a commercially available, Food and Drug Administration-approved test kit (PathVysion HER-2 DNA Probe kit; Abbott Molecular, Downers Grove, IL, USA). The hybridization mixture included a centromere 17-specific green-labeled DNA probe and HER2/neu-specific orange-labeled DNA probe.

The slides coated with whole nuclei were dried in an oven at 65°C for 1 h, and fixed with methanol-glacial acetic acid (3:1) for 1 h. After air drying, the slides were placed in citrate buffer (pH 6.0) and incubated for 10 min in a microwave oven, then transferred to freshly prepared 0.4% pepsin solution (0.16 g pepsin, 2,850 U/mg solid, in 40 ml of 0.9% sodium chloride, pH 1.5) and dehydrated through a series of graded ethanol solutions. After dehydration, 10 μl HER2/CEN-17 probe mix was applied to the tissue and covered with a coverslip. The sections were placed in a HYBrite instrument (Vysis, Inc., Downers Grove, IL, USA), denatured at 82°C for 10 min, and hybridized at 45°C for 16 h. Following hybridization, the slides were washed in stringent wash buffer briefly at room temperature, and then in a 65°C citrate buffer solution for 10 min. The slides were dehydrated, air-dried and counterstained with 4′,6-diamidino-2-phenylindole.

The samples were analyzed under a ×60 oil immersion objective using an Olympus BX61 fluorescence microscope (Olympus, Tokyo, Japan) with the appropriate filters.

FISH signals were assessed by two independent assessors examining 30 non-overlapping nuclei for each sample. The number of chromosome 17 signals and HER2 signals was recorded for each case. Calculation of the mean number of HER2 signals, CEP17 signals and the HER2/CEP17 ratio was performed. HER2 gene amplification status was classified according to the ASCO/CAP criteria (13). The results were calculated as a HER2/CEP17 ratio. Negative HER2 gene amplification was defined as a HER2/CEP17 ratio of <1.8. Equivocal HER2 gene amplification was defined as a HER2/CEP17 ratio between 1.8 and 2.2. Positive HER2 gene amplification was defined as a HER2/CEP17 ratio of ≥2.2.

An average count of chromosome 17 ≥2.6 per nucleus was considered as polysomy (19).

### IHC

A tissue microarray was constructed from the 109 FFPE breast cancer tissue blocks according to a previously described procedure (20). Four tissue cores were selected from the defined regions to construct a TMA in a 30×30 matrix with 16 cores in the last row as a location indicator. Sections (3 μm thick) were cut from the TMA blocks for morphological observations and IHC staining.

The TMA slides were stained with polyclonal rabbit anti-human c-erbB-2 oncoprotein antibody (A0485; DakoCytomation, Carpinteria, CA, USA), using the standard streptavidin-biotin complex method (21), with appropriate positive and negative controls, according to the manufacturer’s instructions. HER2 immunoreactivity was interpreted based on the new ASCO/CAP recommendations (3) and scored as 0 (no staining), 1+ (weak and incomplete membrane staining), 2+ (strong, complete membrane staining in ≤30% of tumor cells or weak/moderate heterogeneous complete membrane staining in ≥10% of tumor cells) or 3+ (strong, complete, homogeneous membrane staining in >30% of tumor cells).

### Statistical analysis

The χ^2^ test was used for nonparametric data (chromosomal ploidy status, HER2 gene amplification, HER2 protein expression, nuclear atypia and lymph node metastasis). SPSS software, version 13.0 (SPSS, Inc., Chicago, IL, USA) was used for statistical analysis. P<0.05 was considered to indicate a statistically significant difference.

## Results

### FISH and IHC analyses

**Figure 1:**
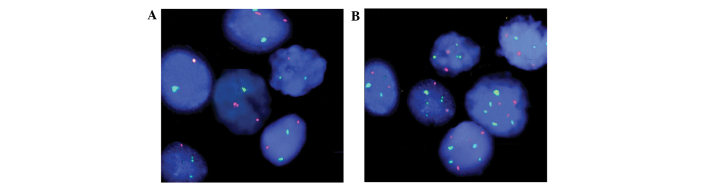
FISH results (A) without or (B) with chromosome 17 polysomy (magnification, ×600). FISH, fluorescence *in situ* hybridization.

**Figure 2:**
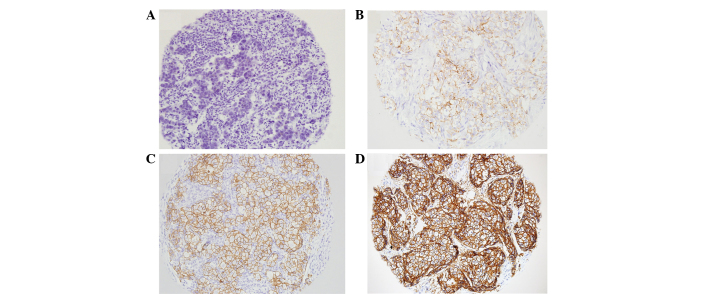
HER2 protein expression detected by IHC (magnification, ×200). (A) Negative, (B) 1+, (C) 2+ and (D) 3+ HER2 expression. HER2, human epidermal growth factor receptor 2; IHC, immunohistochemistry.

The CEP17 and HER2 statuses evaluated by WNFISH were available for all 109 samples. Among the 109 cases, the WNFISH method detected HER2 amplification (HER2 ratio ≥2.2) in 30 cases, equivocal amplification (ratios between 1.8 and 2.2) in 19 cases, and no HER2 amplification (ratio <1.8) in 60 cases. The WNFISH method detected CEP17 polysomy (CEP17 number ≥2.6) in 37 cases and no polysomy (CEP17 number <2.6) in 72 cases. Polyploidy was associated with tumor cell pleomorphism and HER2 gene variability, as detected in the FISH analysis (Fig. 1). Among the 109 cases analyzed by TMA and IHC, 31 cases were found to be HER2-negative, 14 cases were scored 1+, 23 cases were scored 2+ and 41 cases were scored 3+. Representative images of IHC staining for each score are shown in Fig. 2.

### Chromosome 17 polysomy and HER2 gene amplification

Among the 37 cases with chromosome 17 polysomy, HER2 gene amplification was detected in 18 cases (48.6%), while HER2 gene amplification was detected in only 12 cases (16.7%) among the 72 cases without chromosome 17 polysomy (P=0.002; Table I).

### Chromosome 17 polysomy and HER2 protein expression

The correlation between chromosome 17 polysomy and HER2 protein expression is shown in Table I. Among the 37 cases with chromosome 17 polysomy, 3+ HER2 expression was detected in 21 cases (56.8%), while 3+ HER2 expression was detected in only 20 cases (27.8%) among the 72 cases without chromosome 17 polysomy (P=0.003).

### Chromosome 17 polysomy, nuclear atypia and lymph node metastasis

The correlation between chromosome 17 polysomy and nuclear atypia and lymph node metastasis is shown in Table II. In 70.3% (26/37) of the breast cancer cases with polyploidy, the cancer cells had large, unusual or megakaryocytic nuclei with a high degree of nuclear atypia, while no nuclear atypia was observed in 41.7% (30/72) of the breast cancer cases without polyploidy (P=0.008). In cases with chromosome 17 polysomy, the incidence of lymph node metastasis was 73.0% (27/37), while the incidence of lymph node metastasis was only 36.1% (26/72) in cases without chromosome 17 polysomy (P<0.001).

## Discussion

The incidence of chromosome 17 polysomy in invasive breast carcinoma is ~20–40% (10). Watters *et al* (22) reported that cancer cells in ~50% of invasive breast carcinoma cases are aneuploid. This genetic change may be associated with poor prognosis in certain breast cancer patients. In that study, 75% of chromosome 17 polysomy cases were accompanied by HER2 gene amplification. Risio *et al* (23) observed that patients with chromosome monomers and HER2 gene amplification may not respond to trastuzumab treatment. Hammock *et al* (24) demonstrated that a 3+ IHC result without HER2 gene amplification, which is observed in ~50% of patients, was caused by chromosome 17 polysomy. However, the authors identified there was no marked correlation between IHC 2+ and polysomy. A number of multicenter studies (8,25,26) have demonstrated that there was an evident correlation between IHC 3+ and HER2 gene amplification and the sensitivity of patients to targeted therapy. Cases with negative FISH results and IHC 3+ were defined as ‘central HER2-negative’ and mRNA *in situ* hybridization was performed on these cases, identifying that the positive rate of HER2 mRNA was low and confirming that the ‘central HER2-negative’ cases were truly HER2-negative ones.

Thin tissue section FISH is the routinely used method for the detection of gene status in paraffin-embedded breast cancer samples. The average thickness of the sections is 4 μm, which is less than the average diameter of a cell nucleus (6 μm). Thus, in the majority of instances, only a portion of a given nucleus is observed in the tissue sections. Thus, sectioning the tissues into 4-μm-thick sections may cause the loss of nuclear integrity, which may ultimately lead to detection bias in a FISH assay. In the present study, in order to preserve the structure and genetic material of the cells, whole, intact nuclei were extracted from the paraffin-embedded tissues for FISH analysis, and the HER2 gene and CEP17 statuses were detected. It may be assumed that the results obtained through this approach accurately identify the gene status for better predicting the prognosis and response to targeted therapy of patients.

The results show that there was an increase in HER2 gene amplification and HER2 protein expression in the cases with chromosome 17 polysomy, demonstrating that HER2 overexpression in certain breast carcinoma cases may be due to increases in the HER2 gene copy number caused by chromosome 17 amplification. Among the 109 breast cancer specimens assessed using FISH, 37 cases (33.9%) were revealed to have chromosome 17 polysomy. This number was smaller than that in the study by Watters *et al* (22). In the present study, the proportion of cases with chromosome 17 polysomy was not greater than that previously observed, despite the fact that intact nuclei were used for the FISH analysis. The cause of this discrepancy may be due to the small sample size or the different source of samples selected for analysis in the present study.

In the cases with chromosome 17 amplifications, the incidence of nuclear atypia and lymph node metastasis was higher than that in the cases without chromosome 17 amplification. This result indicates that chromosome 17 amplification may correlate with poor prognosis in certain breast cancer patients, which is in agreement with several other studies (13,22). However, further studies are required to investigate the correlation between chromosome 17 amplification and the efficacy of targeted therapy in breast cancer.

Additional studies should aim to explore the associations between chromosome 17 polysomy and the follow-up data of patients after trastuzumab treatment. These future studies may provide information on the efficacy and drug resistance to molecular targeted therapy observed in certain breast cancers.

In conclusion, performing HER2 FISH analysis on whole, intact nuclei from paraffin-embedded tissues is feasible, and provides results correlating with those of IHC. Chromosome 17 polysomy correlates with nuclear atypia and with a higher rate of lymph node metastases.

## Figures and Tables

**Table I:** Correlation between chromosome 17 polysomy and HER2 gene amplification and HER2 protein expression.

Polysomy 17	N	HER2 amplification status			P-value	HER2 protein expression				P-value
	
		Non-amplified^a^	Equivocal^b^	Amplified^c^		−	+	2+	3+	
+	37	15	4	18	0.002	3	4	9	21	0.003
−	72	45	15	12		28	10	14	20	

a HER2/CEP17 ratio <1.8;

b HER2/CEP17 ratio ≥1.8, <2.2;

c HER2/CEP17 ratio ≥2.2.

HER2, human epidermal growth factor receptor 2; CEP17, chromosome 17 centromere.

**Table II:** Correlation between chromosome 17 polysomy and nuclear atypia and lymph node metastasis

Polysomy 17	N	Nuclear atypia		P-value	Metastasis		P-value
	
		Low-grade	High-grade		+	−	
+	37	11	26	0.008	27	10	0.0005
−	72	42	30		26	46	
